# Dual-Drug Delivery Systems Using Hydrogel–Nanoparticle Composites: Recent Advances and Key Applications

**DOI:** 10.3390/gels11070520

**Published:** 2025-07-03

**Authors:** Moon Sup Yoon, Jae Min Lee, Min Jeong Jo, Su Jeong Kang, Myeong Kyun Yoo, So Yeon Park, Sunghyun Bong, Chan-Su Park, Chun-Woong Park, Jin-Seok Kim, Sang-Bae Han, Hye Jin Lee, Dae Hwan Shin

**Affiliations:** 1 College of Pharmacy, Chungbuk National University, Cheongju 28160, Republic of Korea; dy5393@gmail.com (M.S.Y.); lcxowk6@gmail.com (J.M.L.); rkdtnwjd20@gmail.com (S.J.K.); ymk990825@gmail.com (M.K.Y.); psy5266@chungbuk.ac.kr (S.Y.P.); bsh122344@gmail.com (S.B.); cpark@chungbuk.ac.kr (C.-S.P.); cwpark@chungbuk.ac.kr (C.-W.P.); shan@chungbuk.ac.kr (S.-B.H.); 2 Department of Pharmaceutical Chemistry, University of Kansas, Lawrence, KS 66047, USA; jmj950912@gmail.com; 3 Drug Information Research Institute (DIRI), College of Pharmacy, Sookmyung Women’s University, Seoul 04310, Republic of Korea; jsk9574@sookmyung.ac.kr

**Keywords:** dual-drug delivery, polymer-based hydrogels, hydrogel–nanoparticle composites, synergistic therapeutic effects, controlled drug release

## Abstract

Dual-drug delivery systems using hydrogel–nanoparticle composites have emerged as a versatile platform for achieving controlled, targeted, and efficient delivery of two distinct therapeutic agents. This approach combines the high loading capacity and tunable release properties of hydrogels with the enhanced stability and targeting ability of nanoparticles, providing synergistic benefits in various biomedical applications. While significant progress has been made, previous research has primarily focused on single-drug systems or simple co-delivery strategies, often lacking precise spatial and temporal control. This gap underscores the need for more sophisticated composite designs that enable programmable, multi-phase release. This review discusses representative fabrication methods, including physical embedding, covalent integration, and layer-by-layer assembly, to offer insights into practical implementation strategies. Also we present recent studies focusing on key applications—including wound healing, cancer therapy, infection prevention, transplant immunosuppression, and tissue regeneration—with an emphasis on composite design and formulation strategies, types of hydrogels and nanoparticles, and mechanisms of dual-drug release and evaluation. Recent advances in nanoparticle engineering and hydrogel formulation have enabled precise control over drug release and improved therapeutic outcomes. Dual-drug delivery systems using hydrogel–nanoparticle composites present a promising approach for overcoming the limitations of conventional monotherapy and achieving synergistic therapeutic effects. Ongoing research continues to optimize the design, efficacy, and safety of these systems, paving the way for their clinical translation.

## 1. Introduction

### 1.1. Importance of Dual-Drug Delivery

Monotherapy with a single drug has demonstrated moderate efficacy in various diseases; however, the pathological complexity of conditions such as cancer, chronic infections, and immune disorders often limits the therapeutic potential of single-agent regimens. Challenges such as multidrug resistance, compensatory signaling pathways, and divergent pharmacokinetic profiles necessitate more sophisticated delivery strategies [1,2,3,4]. In this context, dual-drug delivery systems have garnered increasing attention due to their capacity to deliver two therapeutics either simultaneously or sequentially in a controlled manner [5,6,7].

Dual-drug delivery systems enable the co-administration of therapeutics with distinct mechanisms of action. For instance, in oncology, the co-delivery of a cytotoxic drug and an autophagy inhibitor can induce synergistic apoptosis, while in infection-related wound healing, concurrent delivery of an antimicrobial and an anti-inflammatory agent can accelerate tissue regeneration while controlling infection [8,9]. This approach is particularly significant in diseases involving multi-target pathophysiology, such as solid tumors with heterogeneous microenvironments or in transplantation immunology, where immunosuppressants and anti-inflammatory agents must be co-localized at the graft site [10,11,12].

Moreover, dual-drug delivery systems offer a strategic advantage in circumvention of drug resistance. Cancer cells, for instance, can frequently develop resistance to single-agent therapies via genetic alteration and clonal selection [13,14]. A dual-drug delivery strategy, especially one combining small molecules with siRNAs or gene modulators, can act via orthogonal pathways to suppress resistance mechanisms at both the transcriptional and post-translational levels [15,16,17,18]. Thus, such systems represent a cornerstone of emerging multi-hit precision medicine strategies.

Importantly, dual-drug delivery systems offer a key advantage by enabling independent control over pharmacokinetics and pharmacodynamics of therapeutic agents, capabilities often unattainable with simple mixtures or conventional dosage forms [19,20,21]. A major strength of these systems lies in the potential for the spatiotemporal control of drug release, particularly when using stimuli-responsive matrices such as pH-, temperature-, or redox-sensitive hydrogels [22,23,24,25,26,27]. These systems ensure that each drug is released only under specific physiological conditions, minimizing unwanted drug–drug interactions. Nanoparticles embedded within hydrogels are especially well-suited for this task, providing compartmentalized, responsive release profiles while also protecting labile drugs from premature degradation [28,29].

In summary, dual-drug delivery systems represent a transformative approach in modern pharmaceutics. They move beyond the scope of simple co-formulation and aim to construct functionally differentiated, programmable delivery platforms. Hydrogel–nanoparticle composites, with their synergistic material properties, have emerged as ideal carriers in this domain. This review will discuss the architectural design, mechanistic release strategies, and application-specific innovations in these platforms, highlighting their potential as the next-generation therapeutic standard.

### 1.2. Need for Hydrogel–Nanoparticle Composites

The therapeutic advantage of dual-drug delivery systems stems not simply from the co-administration of two therapeutic agents, but from the precise and independent control of each agent’s release profiles, spatial localization, and pharmacokinetic behaviors [30,31,32,33]. Achieving such a level of sophistication requires advanced, multifunctional, and structurally dynamic delivery platforms. Hydrogel–nanoparticle composites uniquely meet these demands and have emerged as one of the most promising delivery platforms by integrating the tunable, stimuli-responsive properties of hydrogels with the compartmentalized loading and controlled release features of nanoparticles [34,35,36].

Hydrogels, due to their high water content, biocompatibility, and tunable mesh networks, have long served as attractive scaffolds for drug delivery [37,38]. However, their limitations in encapsulating hydrophobic agents, poor mechanical strength, and limited stimulus-responsiveness restrict their application in advanced dual-drug delivery systems [39,40]. To overcome these drawbacks, researchers have incorporated functionalized nanoparticles within hydrogel matrices or chemically crosslinked them to generate composite systems that synergize the strengths of both materials [41,42,43].

Nanoparticles offer several key advantages, including high drug loading capacity, targeting capabilities via surface modification, and—importantly—stimuli-responsive behavior. By engineering nanoparticles to respond to endogenous signals such as pH shifts, reactive oxygen species (ROS), enzymatic activity, or temperature gradients, researchers can achieve site-specific and condition-triggered drug release [44,45,46,47]. This feature is especially beneficial for dual-drug delivery systems, where controlled, sequential, or staggered release profiles are essential for maximizing therapeutic synergy and minimizing off-target effects.

The hydrogel–nanoparticle composite thus acts as a multi-compartmental system: the hydrogel provides a hydrated, diffusion-controlled bulk environment for one drug, while the nanoparticle subdomain facilitates the protected, stimulus-sensitive delivery of a second agent. Such constructs allow for orthogonal release dynamics, a key requirement when managing drugs with differing physicochemical properties or pharmacological targets [48,49,50]. For instance, an antibiotic can be loaded in the hydrogel for rapid burst release, while an anti-inflammatory agent is embedded in the nanoparticle for slow, sustained delivery [51,52].

Beyond binary encapsulation, these composites enable precise spatiotemporal modulation of drug distribution. Through hierarchical structuring, such as core–shell or layer-by-layer assembly, therapeutic agents can be strategically positioned for phase-specific, sequential, or localized release [53,54]. Moreover, the injectability or in situ gelling properties of these composites facilitate site-specific placement, allowing the formation of therapeutic gradients within tumor margins, inflamed tissues, or graft sites—regions where spatially controlled exposure is critical for maximizing efficacy, reducing systemic toxicity, and enhancing tissue regeneration or immune modulation [55,56,57].

Recent advances have expanded the materials repertoire for these systems. The incorporation of mesoporous silica, metal–organic frameworks (MOFs), carbon nanomaterials, and nanoclays within hydrogels has introduced novel functionalities including photothermal conversion, redox buffering, and enzyme-mimetic catalysis [58,59,60,61,62,63]. Additionally, gold or iron oxide nanoparticles enable thermally triggered release or imaging-guided therapy, augmenting the multifunctionality of the composite system [64,65].

In conclusion, hydrogel–nanoparticle composites represent a structurally integrated platform with the potential to address key challenges in dual-drug delivery, including selective drug loading, controlled and sequential release, biocompatibility, and targeting specificity. While further investigations into scalability, long-term toxicity, and regulatory compliance are necessary to support clinical translation, these systems continue to evolve as promising candidates for advanced combination therapies and precision medicine.

## 2. Design and Fabrication Strategies of Hydrogel–Nanoparticle Dual-Drug Delivery Systems

### 2.1. Composite Formation Methods

#### 2.1.1. Physical Embedding

The most straightforward and widely employed strategy for integrating nanoparticles into hydrogel matrices is physical embedding, wherein pre-formed nanoparticles are dispersed throughout the hydrogel network via non-covalent interactions [66,67]. This method benefits from operational simplicity, scalability, and minimal chemical modification of components, making it especially attractive for drug delivery systems requiring modular design and rapid prototyping [68,69].

In physical embedding approaches, the hydrogel precursor solution is mixed with nanoparticle suspensions, followed by in situ gelation under mild conditions (e.g., temperature, pH, ionic strength). The resulting structure effectively traps nanoparticles within the polymeric meshwork, either through entropic confinement or weak intermolecular forces such as hydrogen bonding, van der Waals interactions, or electrostatic interactions, depending on the surface properties of both the hydrogel and the nanoparticles [70,71].

This method is particularly advantageous in dual-drug delivery systems because it enables spatial segregation of drugs: one drug can be freely dispersed in the hydrogel matrix for rapid release, while another is encapsulated within the embedded nanoparticles for delayed or stimulus-triggered release (Table 1) [72]. For example, hydrophilic antibiotics such as vancomycin may be loaded directly into a hydrogel phase, while hydrophobic anti-inflammatory drugs like curcumin or dexamethasone can be pre-loaded into polymeric or lipid-based nanoparticles before incorporation [73,74]. This compartmentalized loading facilitates orthogonal release profiles and synergistic therapeutic effects.

Another key advantage of physically embedding nanoparticles within hydrogel is preservation of their functional integrity. Since the nanoparticles are not chemically modified during incorporation, their surface ligands, charge characteristics, and responsiveness to stimuli (e.g., pH or ROS) remain intact, allowing for precise control over drug release behavior post-administration. This is particularly important for responsive nanocarriers like mesoporous silica nanoparticles (MSNs), micelles, or dendrimers that rely on internal structure or porosity to regulate drug release [75,76].

However, the method is not without limitations. One major challenge is the heterogeneous distribution of nanoparticles during gelation, particularly in bulk hydrogels, which can lead to uneven drug release. Strategies to mitigate this include pre-crosslinking of nanoparticles, use of shear-thinning hydrogels, or incorporating microscale confinement techniques such as microfluidic mixing [77,78,79]. Another issue is leaching of nanoparticles from loosely crosslinked hydrogels, which may result in premature drug release or reduced targeting efficiency. In such cases, increasing the hydrogel’s mesh density or using physical entrapment-enhancing strategies—such as thermogelling or ionic crosslinking—can improve retention [80,81,82].

Recent examples illustrate the successful use of this method in clinically relevant settings. A 2024 study by Xu et al. utilized a NapFFKK-based peptide hydrogel physically embedded with rapamycin-loaded micelles for corneal graft immunosuppression, showing temporally distinct release kinetics and improved therapeutic outcomes [83]. Similarly, composite gelatin hydrogels embedded with silver-doped nanoparticles have been shown to provide dual antibacterial and anti-inflammatory effects in wound healing models [84].

In summary, physical embedding provides a versatile, modular, and biocompatible route to developing hydrogel–nanoparticle composite systems. While chemical stability and uniform distribution remain concerns, ongoing advances in nano–hydrogel interactions and gelation dynamics are progressively mitigating these limitations, making this approach a cornerstone of next-generation dual-drug delivery design.

#### 2.1.2. Covalent Integration

While physical embedding offers simplicity and biocompatibility, covalent integration provides enhanced structural stability and precise control over drug release profiles, making it a critical design strategy for advanced hydrogel–nanoparticle composite systems. In this approach, nanoparticles are chemically bonded to the hydrogel matrix, forming a robust and functionally cohesive platform that excels in mechanical strength, in vivo retention, and sustained delivery [85,86]. This is particularly advantageous for dual-drug delivery systems, where physical separation and independent control of two therapeutic agents are essential (Table 1).

Covalent integration is typically achieved through two main routes: (1) by introducing reactive functional groups (e.g., -NH_2_, -COOH, -SH) onto the nanoparticle surface, which can then participate in crosslinking during hydrogel polymerization, or (2) by incorporating pre-functionalized polymers into the hydrogel backbone that can react with nanoparticles post-formation [87,88]. The chemical reactions employed often include Michael addition, Schiff base formation, isocyanate–amine coupling, and various “click” chemistries, notably azide–alkyne cycloaddition, all of which can proceed under mild, biocompatible conditions [89,90,91,92].

One of the primary advantages of covalent integration is positional stability. Unlike physically embedded nanoparticles, which may leach out over time, covalently anchored nanoparticles become an integral part of the hydrogel’s crosslinked network, ensuring consistent therapeutic localization. This is particularly vital for potent agents such as immunosuppressants or anticancer drugs, where premature release or systemic exposure must be minimized [93].

Covalent attachment also enables multimodal and hierarchical drug release design. The hydrogel matrix can be responsible for rapid or sustained release of one drug via passive diffusion, while the nanoparticle domain—covalently tethered—can respond selectively to pH, ROS, enzymatic activity, or other stimuli to trigger the secondary release. This architecture facilitates orthogonal kinetics, ideal for scenarios requiring staggered pharmacological effects [94,95].

However, this strategy is not without challenges. The chemical reactions involved often require careful optimization of reaction conditions, purification of byproducts, and compatibility with sensitive therapeutics. For biomacromolecules like peptides, proteins, or RNA, covalent methods may risk loss of activity or structural denaturation [96,97]. In such cases, the use of bio-orthogonal chemistries—highly selective, non-toxic reactions such as strain-promoted azide–alkyne cycloaddition (SPAAC)—has gained popularity due to their efficiency and mildness in physiological environments [89].

Recent studies exemplify the application of this method in dual-delivery systems. Lin et al. utilized Dialdehyde-functionalized Pluronic F127 micelles, which were covalently crosslinked with a phenol-modified chitosan hydrogel (CSH) through Schiff base reactions, providing structural stability. In the context of anticancer therapy, the hydrophilic drug minocycline was loaded into the hydrogel matrix, enabling a rapid initial release, while the hydrophobic drug edaravone was encapsulated within the micellar core, allowing for sustained secondary release [98].

Another notable example involves isocyanate-functionalized polyurethane hydrogels covalently linked to micelles encapsulating insulin and GLP-1 analogs. The resulting dual-drug delivery system exhibited improved glycemic control and β-cell protection in diabetic models, highlighting its potential for metabolic disease management through spatiotemporally controlled delivery [99,100].

In summary, covalent integration offers a highly stable, customizable, and precise strategy for engineering hydrogel–nanoparticle composites tailored to dual-drug delivery. As the complexity of therapeutic regimens increases, these systems are expected to play a central role in personalized combination therapies and next-generation biomedical engineering platforms.

#### 2.1.3. Layer-by-Layer Assembly

Layer-by-layer (LbL) assembly is a bottom-up fabrication strategy that creates multilayered structures by alternately depositing materials layer-by-layer. Over the past few decades, this method has proven to be simple, cost-effective, and highly versatile, enabling precise control over composition and architecture at the nanometer scale [101,102].

In an LbL-assembled hydrogel–nanoparticle composite, layers of polymers (including hydrogel-forming polyelectrolytes) and nanoparticle components are built up in an alternating fashion. Each adsorption step deposits a thin layer of material through complementary interactions, typically by exploiting electrostatic attraction between oppositely charged species. Other interactions—such as hydrogen bonding, covalent bonding, or specific affinity interaction—can also be utilized to bind layers together [103]. This layer-by-layer approach allows integration of diverse building blocks—ranging from polymers and biomacromolecules to inorganic nanoparticles—within a single composite film (Table 1).

LbL multilayers can be fabricated on various substrates (including hydrogel surfaces) using different deposition techniques. The traditional method is dip-coating (sequential immersion of the substrate in alternating solutions) [104], but newer approaches like spray-coating and spin-coating are also widely used [105,106]. Each technique offers distinct advantages: dipping ensures thorough layer formation even on irregular or porous surfaces, whereas spraying and spinning allow faster, more uniform layering on planar substrates [107]. Spray deposition tends to produce thinner, smoother layers with potentially higher drug loading compared to dipping, while spin-coating can yield films with reduced roughness and better uniformity than dip-coating [108].

In the context of dual-drug delivery, LbL assembly offers precise control by physically compartmentalizing two therapeutic agents into distinct layers or domains [109]. This multilayer design can keep different drugs separated and protected—for example, one drug may be loaded in a hydrogel core or a specific polymer layer while another is confined to a nanoparticle-containing outer shell [110]. Such spatial segregation minimizes direct drug–drug interactions and allows each compound’s release kinetics to be tuned independently via the properties of its respective layer (e.g., layer composition, degradability, or stimuli-responsiveness).

Multilayer LbL films have accordingly been developed to carry multiple drugs with the ability to program sequential or on-demand release profiles. By adjusting the layer order and composition (for instance, inserting a nanoparticle-rich barrier layer), one can modulate overall release rates and achieve staged release of the two agents [111]. LbL assemblies can be implemented on hydrogel supports (e.g., coating a bulk hydrogel or microgel with drug-loaded layers) or fabricated as free-standing films/capsules that themselves incorporate hydrogel and nanoparticle components [112,113]. In both formats, the layered architecture creates separate “compartments” for each drug, allowing each payload to be released in a controlled, programmed manner.

- Physical compartmentalization: Each drug is confined to separate layers or zones, preventing interference and allowing distinct microenvironments optimal for each (e.g., a hydrophobic drug in a nanoparticle layer versus a hydrophilic drug in a hydrogel layer) [114].
- Tailored release kinetics: By adjusting layer composition and thickness, the release rate of each drug can be independently tuned—even enabling sequential or staged release for one drug before the other [115].
- Multifunctional versatility: LbL films can integrate many material types (polymers, inorganic nanoparticles, biomolecules, etc.), making it feasible to co-deliver drugs with very different properties or activation triggers within one composite [116].

**Table 1:** Comparison of formulation strategies for hydrogel–nanoparticle dual-drug delivery systems: Key features, advantages, and drawbacks.

Formulation Strategy	Key Features	Advantages	Drawbacks
Physical Embedding	Pre-formed nanoparticles are physically dispersed within the hydrogel matrix via non-covalent interactions	- Simple fabrication - Benefits of modular design and rapid prototyping - Preserving hydrogel and nanoparticle functionality	- Non-uniformity of nanoparticle distribution - Release control limitations
Covalent Integration	Nanoparticles are chemically bonded to the hydrogel network (e.g., via EDC/NHS or click chemistry)	- Positional stability - Multimodal and hierarchical drug release design	- Requires precise reaction control and purification - Risk of activity loss or denaturation for sensitive therapeutics
Layer-by-Layer Assembly	Multilayer structures are sequentially deposited on hydrogel or nanoparticle surfaces	Spatially separated dual-drug release control	- Limited formulation flexibility in multilayer architectures - Restricted drug loading per layer

### 2.2. Types of Hydrogels

#### 2.2.1. Natural Polymers

Natural polymer-based hydrogels have long been valued for their intrinsic biocompatibility, biodegradability, and bioactivity, making them attractive for biomedical applications including dual-drug delivery. Representative materials include chitosan, gelatin, and alginate, each offering distinct physicochemical properties suitable for constructing hydrogel–nanoparticle composite systems [117].

Chitosan, a cationic polysaccharide, contains primary amine groups that facilitate electrostatic interactions with negatively charged drugs (e.g., nucleic acids) and nanoparticles. This property makes it highly suitable for compartmentalizing drugs in dual-delivery platforms. In particular, chitosan exhibits pH-sensitive solubility, enabling site-specific release in acidic conditions such as tumor microenvironments or the gastrointestinal tract [118,119].

Gelatin, a collagen-derived polypeptide, offers excellent cell adhesion and tissue compatibility, making it especially useful for regenerative medicine-oriented drug delivery systems (DDSs). It can be thermally gelled to form temperature-sensitive matrices and is amenable to physical or chemical incorporation of nanoparticles such as silver, gold, or liposomes [120].

Alginate, an anionic polysaccharide, rapidly gels upon exposure to divalent cations (e.g., Ca^2+^, Ba^2+^), forming ionically crosslinked networks. This property, combined with its excellent muco-adhesiveness and tunable porosity, makes alginate particularly beneficial for dual-drug delivery systems. Its core–shell structuring capacity makes it attractive for encapsulating nanoparticles in multilayered DDSs. However, the resultant gels are mechanically weak and may offer limited nanoparticle retention, necessitating structural enhancements [121].

Despite their favorable biocompatibility and bioactivity, natural polymer-based hydrogels often present critical limitations that hinder their standalone use in advanced dual-drug delivery systems. Chitosan exhibits poor water solubility and limited mechanical strength, necessitating chemical crosslinking agents such as genipin or glutaraldehyde to form stable hydrogels [118]. Gelatin is highly susceptible to enzymatic degradation, leading to rapid breakdown under physiological conditions; thus, structural reinforcement strategies—such as double-network designs—are often required to maintain drug release control [122]. Alginate hydrogels, while easy to gel through ionic crosslinking, are mechanically weak and frequently exhibit poor nanoparticle retention, thereby limiting their ability to support sustained or localized release [123]. These inherent drawbacks underscore the need to reinforce natural polymers with synthetic components or nanoparticles to enhance mechanical integrity, responsiveness, and drug release precision [124].

#### 2.2.2. Synthetic Polymers

Synthetic polymer-based hydrogels are highly attractive for dual-drug delivery systems due to their engineerable network structures, reproducibility, and tunable responsiveness. Key examples include polyethylene glycol (PEG), polyvinyl alcohol (PVA), and poly(N-isopropylacrylamide) (PNIPAM), each offering distinct advantages for integrating nanoparticles and managing complex drug release profiles [125].

PEG is a gold-standard material in biomedicine for its non-immunogenicity and hydrophilicity. Its terminal groups can be easily functionalized, and PEG–diacrylate (PEGDA) hydrogels formed via photopolymerization allow precise fabrication of nanoparticle-embedded matrices. PEG’s high water content and inertness make it ideal for sustained diffusion-controlled drug release, and its compatibility with other biopolymers (e.g., gelatin, hyaluronic acid) supports composite hydrogel design [126,127].

PVA is capable of forming physically crosslinked gels through extensive hydrogen bonding, and at high concentrations, it exhibits excellent mechanical strength and structural stability. Though PVA’s interaction with nanoparticles is relatively limited due to its neutral, hydrophilic nature and lack of functional groups for strong electrostatic or covalent bonding, freeze–thaw cycles can yield chemical crosslinker-free hydrogels, making it useful in non-biodegradable, localized delivery settings [128].

PNIPAM is the archetypal thermoresponsive hydrogel, exhibiting a lower critical solution temperature (LCST) around 32 °C. This temperature-sensitive phase transition allows for thermally triggered drug release, as PNIPAM becomes hydrophobic and contracts above its LCST, expelling water and entrapped drugs. This enables thermal triggering of drug release, and its combination with nanoparticles (e.g., gold, micelles) facilitates dual-stimuli or photothermal-responsive systems. However, due to its limited biodegradability, PNIPAM is often blended with degradable polymers for in vivo applications [129,130].

Synthetic hydrogels surpass natural counterparts in mechanical control, structural customization, and stimulus sensitivity, making them essential materials for engineered, precision dual-drug delivery systems.

### 2.3. Types of Nanoparticles

#### 2.3.1. Polymeric Nanoparticles

Polymeric nanoparticles are among the most versatile and tunable drug carriers, offering benefits such as protection of cargo, sustained release, and chemical functionalization. Common materials include polylactic-co-glycolic acid (PLGA), polylactic acid (PLA), PEG-PLGA block copolymers, and chitosan-based systems [131].

In dual-drug delivery systems, polymeric nanoparticles allow for either co-encapsulation or spatial separation of two drugs via core–shell structures or multilayer assembly. One drug can be embedded within the polymeric matrix, while the second is adsorbed onto the surface or confined in an outer coating layer, thus enabling simultaneous or sequential release profiles [132].

When embedded within hydrogels, polymeric nanoparticles remain well dispersed and protected, and the hydrogel matrix supports diffusion-controlled release. Additionally, stimuli-responsive polymers (pH-, enzyme-, or temperature-sensitive) can be used to design multi-triggered release systems within the hydrogel composite [133].

Limitations include relatively low drug loading capacity compared to liposomes or inorganic NPs, and potential local acidity from degradation byproducts (e.g., lactic acid from PLA) in vivo [134].

#### 2.3.2. Inorganic Nanoparticles (e.g., Gold, Iron Oxide, Silica)

Inorganic nanoparticles are widely used in dual-drug delivery due to their structural stability, surface modifiability, and external stimuli-responsiveness, including photothermal, magnetic, and fluorescent properties.

Gold nanoparticles (AuNPs) provide excellent biocompatibility and strong optical absorption, making them ideal for photothermal-triggered drug release [135]. Iron oxide nanoparticles are responsive to magnetic fields and can be used for magnetically guided delivery or release modulation [136]. MSNs offer a large surface area and tunable pore sizes, supporting dual-drug loading in compartmentalized architectures [137].

Within hydrogel matrices, inorganic nanoparticles can act as triggering agents—for example, AuNPs can locally heat a thermoresponsive hydrogel upon near-infrared (NIR) exposure, inducing controlled release [138]. However, challenges include a lack of biodegradability and potential long-term toxicity, especially for silica or metallic oxides, requiring immobilization or surface modification strategies to mitigate risks [139].

#### 2.3.3. Liposomes

Liposomes are amphiphilic vesicular carriers composed of phospholipid bilayers capable of encapsulating both hydrophilic (aqueous core) and hydrophobic (lipid bilayer) drugs. Their structural similarity to biological membranes grants them excellent biocompatibility and facilitates efficient cellular uptake [140].

For dual-drug delivery, liposomes are particularly effective as they can simultaneously load drugs of differing solubility. Moreover, their surfaces can be easily modified with PEG (PEGylation), targeting ligands, or stimuli-responsive moieties, enabling tailored delivery profiles [141].

Liposomes can be physically entrapped within the hydrogel network during gelation. When embedded within hydrogels, liposomes can remain stably within hydrogels for days to weeks, depending on factors such as crosslinking density, polymer type, and environmental conditions. This is especially useful for localized, subcutaneous, or mucosal drug delivery, where prolonged therapeutic presence and spatially restricted release are desired [142].

However, key challenges include maintaining liposomal stability—fusion, leakage, or degradation can be exacerbated by strong ionic interactions or harsh gelatin conditions. Strong interactions with the hydrogel matrix may also risk structural collapse, necessitating careful optimization of formulation conditions such as pH, ionic strength, and polymer type [143,144].

## 3. Drug Release Mechanisms in Dual-Drug Delivery Systems

### 3.1. Simultaneous vs. Sequential Release

In dual-drug delivery systems, the choice between simultaneous and sequential release strategies is fundamentally shaped by therapeutic goals, drug–drug interactions, and the pathological context of the target tissue. These strategies impose distinct demands on the structural design and material composition of hydrogel–nanoparticle composites [20].

Simultaneous release refers to concurrent delivery of two therapeutic agents within the same time window. This is typically achieved through co-encapsulation in a shared carrier or homogeneous distribution within the composite matrix. It is well-suited for therapeutic combinations that exhibit synergistic effects, such as antibiotic pairs or chemotherapeutics with vascular normalizers. From a formulation standpoint, simultaneous release systems are generally simpler to design and manufacture compared to sequential release platforms, as they do not require complex spatial or temporal compartmentalization [145]. However, simultaneous release may encounter problems when the two drugs possess markedly different physicochemical properties—such as solubility, molecular weight, or stability—which can result in non-uniform release kinetics or premature burst release of one component [146].

In contrast, sequential release involves a time-staggered delivery of two therapeutic agents, often achieved through advanced structuring strategies such as layered architectures, core–shell particles, or stimuli-responsive compartmentalization. This strategy enables one agent—often referred to as a priming drug—to condition the microenvironment before the second agent is released [147]. Classic examples include priming with a chemotherapeutic followed by immunomodulators, or antibiotic administration followed by anti-inflammatories [148,149].

Sequential release systems enable us to mimic natural treatment regimens and biological timing, thereby enhancing therapeutic efficacy while minimizing off-target effects and toxicity. However, achieving reliable sequential release requires more sophisticated structural integration and precise release control than simultaneous systems. In hydrogel–nanoparticle composites, this is often achieved by designing systems where the hydrogel component supports early release, while nanoparticles embedded within the gel provide delayed or stimulus-triggered secondary release. Fine-tuning factors such as polymer degradation rate, crosslinking density, and nanoparticle surface chemistry are essential for achieving predictable kinetics [150,151,152].

In summary, simultaneous release offers simplicity and is ideal for treatments requiring synchronized drug action, whereas sequential release allows for temporal separation of functions, crucial in protocols that rely on preconditioning or multi-step therapeutic cascades. Both strategies can be tailored through intelligent material design and modeling of release kinetics.

### 3.2. Spatial Separation in Composite Structures

In dual-drug delivery systems, spatial separation of therapeutics within the carrier structure is essential for achieving independent release kinetics, site-specific delivery, and minimization of drug–drug interference. In hydrogel–nanoparticle composites, this is commonly realized by physically isolating the two drugs into different structural compartments, enabling multifunctionality within a unified delivery platform [153].

One classical configuration is the double-matrix or core–shell architecture, where a hydrophilic drug is loaded into the hydrogel matrix and a hydrophobic drug is encapsulated in nanoparticles. This allows each drug to reside in a distinct physicochemical environment, resulting in tailored release profiles and improved stability. The hydrogel and nanoparticle domains thus serve as functionally independent reservoirs [154].

Advanced strategies include layer-by-layer assembly, micropatterning, and compartmentalized microcapsules, which allow nanometer- to micrometer-scale localization of different drugs [155,156,157]. In microgel-based systems, for instance, Drug A may be localized near the periphery for rapid diffusion, while Drug B is embedded deeper within the particle for delayed release [158].

Beyond release control, spatial separation also supports biological compartmentalization. Toxic agents can be confined to tumor tissues, while immunomodulators are directed to lymphoid organs, thereby enhancing therapeutic precision and reducing systemic toxicity [159].

In summary, spatial separation is a foundational design principle in hydrogel–nanoparticle dual-delivery systems, enabling distinct functional pathways through intelligent microstructural compartmentalization and materials engineering.

### 3.3. Stimuli-Responsive Release Control

#### 3.3.1. pH-Responsive Release

pH-responsive systems are among the most widely explored stimuli-responsive strategies in dual-drug delivery platforms. These systems exploit the acidic or alkaline microenvironments of pathological sites such as tumors, infected tissues, or intracellular organelles (e.g., lysosomes, endosomes) to trigger selective or sequential drug release [160,161].

In hydrogel–nanoparticle composites, pH responsiveness is typically implemented in two major ways:

First, by using pH-sensitive hydrogel matrices, such as chitosan, poly(β-amino ester), or poly(acrylic acid) [162]. These hydrogels undergo swelling, dissolution, or crosslink degradation in acidic pH, resulting in rapid drug diffusion. In such designs, the hydrogel often facilitates initial burst release, while embedded nanoparticles can mediate secondary sustained release [163].

Second, by incorporating pH-labile linkages within the nanoparticle structure. For example, drugs conjugated via Schiff bases, hydrazones, or orthoester linkers are stable at physiological pH (7.4) but cleaved in mildly acidic environments (pH 5–6), such as endosomes. This enables site-specific release inside targeted cells [164].

pH-triggered systems are widely used to enable stage-wise drug delivery: the hydrogel first degrades under acidic pH to release doxorubicin and curcumin-loaded liposomes, followed by the activation of curcumin-loaded liposomes upon endosomal entry [165].

One of the primary challenges in pH-responsive drug delivery is the narrow pH differential between physiological and pathological environments, which may result in insufficient sensitivity or unintended drug release. Furthermore, pH gradients can vary significantly between patients, tissue types, and disease stages, limiting the universality and predictability of the approach. To address this, pH-responsive systems are often integrated with multi-stimuli designs (e.g., combining with redox or enzyme triggers) for enhanced performance [166].

#### 3.3.2. Temperature-Responsive Release

Temperature-responsive hydrogel–nanoparticle systems enable precise control over the timing and rate of drug release in response to a physiological cue (e.g., local inflammation or fever) or externally applied heat (e.g., near-infrared irradiation or hyperthermia). These systems are particularly useful in dual-drug delivery where thermal triggers can create spatially and temporally selective release zones [167].

A widely used thermoresponsive polymer is PNIPAM, which undergoes a phase transition at LCST around 32 °C. Above this threshold, PNIPAM undergoes volume phase transition, leading to shrinkage of the hydrogel network and modulation of drug diffusion [168]. As such, PNIPAM-based hydrogels can act as thermal switches that respond to body heat or external stimuli (e.g., infrared light, warm compresses) to initiate or halt drug release [169].

In hydrogel–nanoparticle composites, temperature control allows staged release of two drugs. For instance, the hydrogel component may support passive diffusion of 5-fluorouracil at body temperature, while embedded nanoparticles are designed with heat-sensitive linkers or thermodegradable shells, releasing ibuprofen only upon additional thermal stimulation [170].

Moreover, coupling with photothermal nanoparticles (e.g., gold nanorods, carbon nanotubes) allows external light to be converted into localized heat. This facilitates on-demand drug release, where NIR irradiation triggers drug ejection by inducing localized hydrogel shrinkage [171].

However, concerns remain regarding repetitive thermal exposure, which may affect system stability or lead to unintended tissue damage [172]. Thus, development efforts focus on reversible, mild thermal-responsive designs for safe and efficient use in clinical settings.

#### 3.3.3. Enzyme-Responsive Release

Enzyme-responsive release systems leverage disease-specific enzymatic activity to trigger targeted drug delivery. This strategy is particularly advantageous for highly selective dual-drug delivery systems, especially in environments such as tumors, inflamed tissues, or infection sites where specific enzymes are overexpressed [173,174].

Key target enzymes include matrix metalloproteinases (MMPs), hyaluronidases, cathepsins, and cytochrome P450, which are typically abundant in tumor microenvironments or pathological tissue [175,176,177,178]. In hydrogel–nanoparticle composites, these enzymes can cleave peptide-based crosslinkers, biodegradable polysaccharide coatings, or enzyme-sensitive shells, resulting in controlled drug release. In dual-drug applications, enzyme responsiveness can also facilitate sequential release, where each drug is released in response to different enzymatic cues [179,180,181]. For instance, hydrogels conjugated with phenylborate esters can degrade in response to glucose secreted from diabetic wounds, leading to the rapid release of insulin. Gelatin microspheres can be degraded by MMP-9, resulting in the gradual release of encapsulated celecoxib [182].

This strategy offers excellent spatial selectivity and operates via endogenous biological cues. However, limitations include variability in enzyme expression across patients and the risk of off-target leakage [46]. To overcome this, multi-stimuli designs (e.g., combining enzyme with pH or ROS responsiveness) are frequently adopted [183].

#### 3.3.4. Redox or ROS-Responsive Release

Redox and ROS-responsive release systems exploit elevated intracellular glutathione (GSH) levels or ROS commonly found in tumor tissues, inflamed areas, or autoimmune sites. These triggers exist at concentrations up to 100–1000 times higher than in normal tissue, making them powerful cues for pathology-specific drug delivery [184,185].

Redox-responsive systems often utilize disulfide bonds that cleave under high GSH concentrations [186]. These bonds can be incorporated into hydrogel crosslinkers or nanoparticle coatings, enabling structural breakdown and controlled release upon entering the reducing intracellular environment. For instance, nanoparticles coated with GSH-sensitive shells will remain stable in circulation but degrade selectively within target cells [187,188].

ROS-responsive designs rely on chemical motifs such as boronic esters, thioethers, or selenium-based linkers, which degrade in oxidative stress environments characterized by H_2_O_2_, O_2_^−^, or hypochlorite [189]. This allows for triggered drug release in diseased tissues only, reducing off-target toxicity.

In dual-drug delivery systems, one drug can be released by diffusion from the hydrogel, while the second is selectively released from ROS/redox-sensitive nanoparticles [190]. Alternatively, hydrogels crosslinked via disulfide bonds can degrade in response to GSH, releasing both drugs simultaneously in intracellular environments [51].

These systems are especially valuable for intracellular targeting, antioxidant therapy, and tumor-selective cytotoxicity, and are frequently incorporated into multi-stimuli platforms combining redox/ROS triggers with pH or enzyme sensitivity [191,192].

### 3.4. Mathematical Models for Dual-Drug Release

To understand and predict the release behavior of two drugs in a dual-drug delivery system, mathematical modeling is essential [193,194]. In hydrogel–nanoparticle composites, each drug may follow distinct release mechanisms, routes, and kinetics, necessitating models that can capture this complexity [28,195,196].

Commonly used models include the following:

Higuchi model: Assumes drug release is proportional to the square root of time, suitable for diffusion-controlled release from hydrogels.

Korsmeyer–Peppas model: Described by M_t_/M_∞_ = kt^n^, where the exponent n indicates the release mechanism. *n* < 0.5: Fickian diffusion; 0.5 < *n* < 1: anomalous transport; *n* = 1: zero-order.

First-order model: Assumes the release rate is concentration-dependent, often used for degradable nanoparticle systems.

Zero-order model: Ideal model where a drug is released at a constant rate regardless of concentration. Difficult to achieve but targeted in dual-drug designs.

In dual-drug delivery systems, distinct models may be applied to each drug simultaneously, or composite multi-compartment or biexponential models may be used to describe simultaneous and sequential release phases [197,198,199]. For example, one drug may follow Higuchi kinetics (from hydrogel), while the other follows first-order kinetics (from nanoparticles), and their cumulative release is modeled as the sum [200].

Experimental data is typically fitted using MATLAB R2022a (MathWorks, Natick, MA, USA), Origin, or GraphPad Prism (version 8.4), Origin, or GraphPad. Recently, machine learning-based predictive models are also gaining attention in modeling nonlinear or stimulus-responsive systems [201].

However, due to the inherent nonlinearity in swelling hydrogels, nanoparticle disintegration, and stimuli sensitivity, single kinetic models are often insufficient. Hybrid or empirical models are recommended, especially for preclinical simulation and rational design of programmable release profiles [202,203].

## 4. Key Biomedical Applications: Case Studies

### 4.1. Wound Healing

#### 4.1.1. Metformin–Curcumin Compartmentalized Release via Injectable Self-Healing Hydrogel for Diabetic Wounds

An injectable, self-healing hydrogel platform was developed for diabetic wound healing by incorporating dual-drug compartments: mesoporous polydopamine nanoparticles (MPDA NPs) loaded with curcumin, and a hydrogel matrix embedding metformin. The hydrogel was synthesized from N-carboxyethyl chitosan (CEC) and oxidized sodium alginate (OSA), which crosslinked through dynamic Schiff base reactions, enabling self-healing and injectability. MPDA NPs were first fabricated through a templating approach using Pluronic F127 and 1,3,5-trimethylbenzene, followed by dopamine polymerization and subsequent removal of the template to generate high surface area particles capable of hydrophobic drug loading. Curcumin was physically adsorbed into the MPDA NPs via π–π stacking and hydrophobic interactions, while metformin, being hydrophilic, was directly dissolved into the hydrogel precursor solution prior to gelation. Upon subcutaneous injection into diabetic wound sites in mice, the hydrogel formed in situ and provided a biphasic release profile: metformin was released rapidly from the matrix to promote angiogenesis, while curcumin was gradually released from the MPDA over several days to reduce inflammation and oxidative stress. The spatially separated encapsulation strategy avoided drug–drug interference and matched the temporal therapeutic needs of chronic wounds. The system also exhibited ROS-scavenging and hemostatic properties derived from the MPDA NPs, further contributing to wound repair (Table 2) [204].

#### 4.1.2. Curcumin and Ciprofloxacin Dual Loading in Clay-Reinforced Chitosan–Hyaluronic Acid Hydrogel

**Figure 1:**
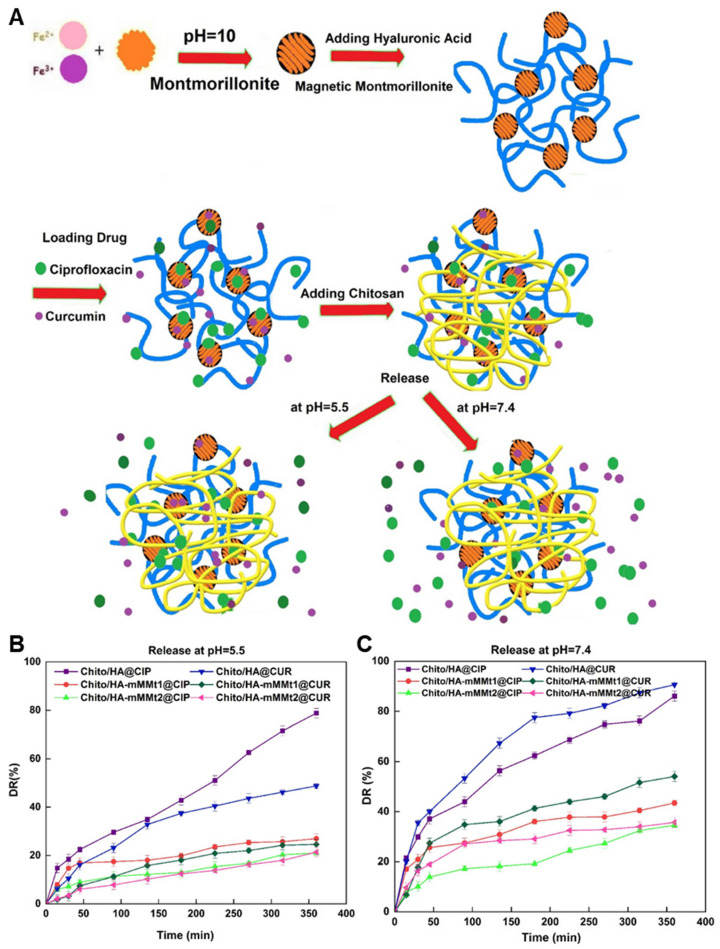
(**A**) Scheme showing the process of synthesizing Chito/HA-mMMt nanocomposite hydrogels. (**B**,**C**) Ciprofloxacin and curcumin release behavior at different pHs. Reprinted with permission from the complete reference citation [205]. Copyright © 2023, BioMed Central.

A dual-drug delivery wound healing system was designed by integrating curcumin and ciprofloxacin into a chitosan/hyaluronic acid hydrogel reinforced with montmorillonite (MMT) nanosheets (Figure 1A). The hydrogel was formed through ionic crosslinking between the carboxyl groups of hyaluronic acid and the amino groups of chitosan, producing a physically stable and bioadhesive network. MMT, a layered silicate clay, was used both to enhance mechanical strength and as a nanocarrier for hydrophobic curcumin via interlayer adsorption. Curcumin was intercalated into the MMT nanosheets by solvent evaporation, while ciprofloxacin, being water-soluble, was incorporated directly into the hydrogel matrix before gelation. This configuration enabled controlled, differential release: ciprofloxacin showed faster diffusion from the hydrogel network to inhibit bacterial proliferation (86.1% at pH 5.5), whereas curcumin was released more slowly from the MMT layers to modulate inflammation and oxidative stress (48.7% at pH 5.5) (Figure 1B,C). The chitosan–hyaluronic acid hydrogel significantly promoted fibroblast migration after 48 h in a scratch assay, demonstrating its potential to enhance wound healing. It also showed strong antibacterial activity, producing the largest inhibition zones against *E. coli* and *S. aureus*. Although the study was not conducted in a chronic wound model, these findings suggest its applicability as a dual-functional wound dressing capable of both infection control and tissue regeneration [205].

#### 4.1.3. Sequential Release of Ciprofloxacin and Insulin from Injectable Hydrogel for Infected Wounds

A dual-drug injectable hydrogel system was developed to address infected wound healing by delivering ciprofloxacin and insulin sequentially through compartmentalized carriers. The hydrogel matrix consisted of oxidized dextran (ODEX) crosslinked with carboxymethyl chitosan (CMCS) via Schiff base formation, enabling in situ gelation and biodegradability. Ciprofloxacin was loaded within a sodium alginate hydrogel network. Insulin was separately loaded into chitosan nanoparticles (CSNPs) through double emulsion–solvent evaporation, then dispersed throughout the hydrogel. Upon subcutaneous injection, the hydrogel solidified locally at the wound site. Ciprofloxacin showed rapid release from sodium alginate hydrogel matrix to exert antibacterial activity during the early infection phase, while insulin was released gradually from CSNPs to support epithelial regeneration and metabolic modulation. SA-Cip/CSNP-INS also contributed by reducing TNF-α levels and increasing TGF-β expression, thereby modulating inflammation. The system demonstrated temporally programmed delivery, minimizing drug interference and aligning drug availability with distinct phases of the wound healing process. This strategy allowed precise kinetic control through distinct nanocarrier domains while maintaining hydrogel integrity and local retention [206].

#### 4.1.4. Layered Hydrogel System for Phase-Specific Release of Curcumin and Pirfenidone

**Figure 2:**
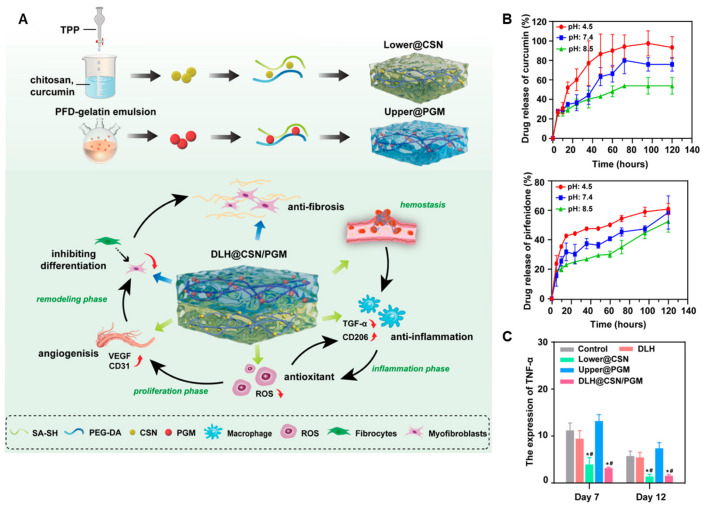
(**A**) Schematic image of synthesis of bilayer hydrogel and sequentially releasing bilayer hydrogel drug delivery system. (**B**) Drug release at different pHs. (**C**) TNF-α fluorescence intensity. Reprinted with permission from complete reference citation [54]. Copyright © 2025, Molecular Diversity Preservation International.

A double-layered hydrogel was engineered to achieve phase-specific wound healing through the sequential release of curcumin and pirfenidone (Figure 2A). The upper layer consisted of a gelatin-based hydrogel incorporating pirfenidone-loaded gelatin microspheres (PGMs), designed to be retained longer and released during the tissue remodeling phase. The lower layer was composed of a polyethylene glycol diacrylate (PEGDA) hydrogel matrix embedded with curcumin-loaded chitosan nanoparticles (Cur@CS-NPs), which degraded faster in early inflammatory environments. The two hydrogel layers were fabricated separately and then combined to form a cohesive structure (double-layer hydrogel drug delivery system (DLH@CSN/PGM)). Cur@CS-NPs were synthesized by ionic gelation, enabling encapsulation of hydrophobic curcumin within the chitosan matrix. PGMs were fabricated using a water-in-oil emulsion method, allowing sustained release of pirfenidone from the upper compartment. Upon application to full-thickness skin wounds, the lower layer released curcumin early to reduce inflammation and scavenge ROS, while the upper layer maintained structural integrity and gradually released pirfenidone to inhibit fibrosis during later stages (Figure 2B). In addition, the DLH@CSN/PGM suppressed the expression of TNF-α, a representative pro-inflammatory cytokine involved in the progression of chronic wounds, suggesting its potential for modulating chronic inflammation during wound healing (* *p* < 0.05 vs. control group; # *p* < 0.05 vs. model group) (Figure 2C). This vertically stratified delivery system aligned drug release kinetics with the wound healing cascade, enhancing regeneration and reducing scar formation without requiring external stimuli or multi-step interventions [54].

#### 4.1.5. Co-Delivery of hMSCs and Antibiotic via GelMA Microparticle–Nanoparticle Hybrid for Enhanced Wound Repair

**Figure 3:**
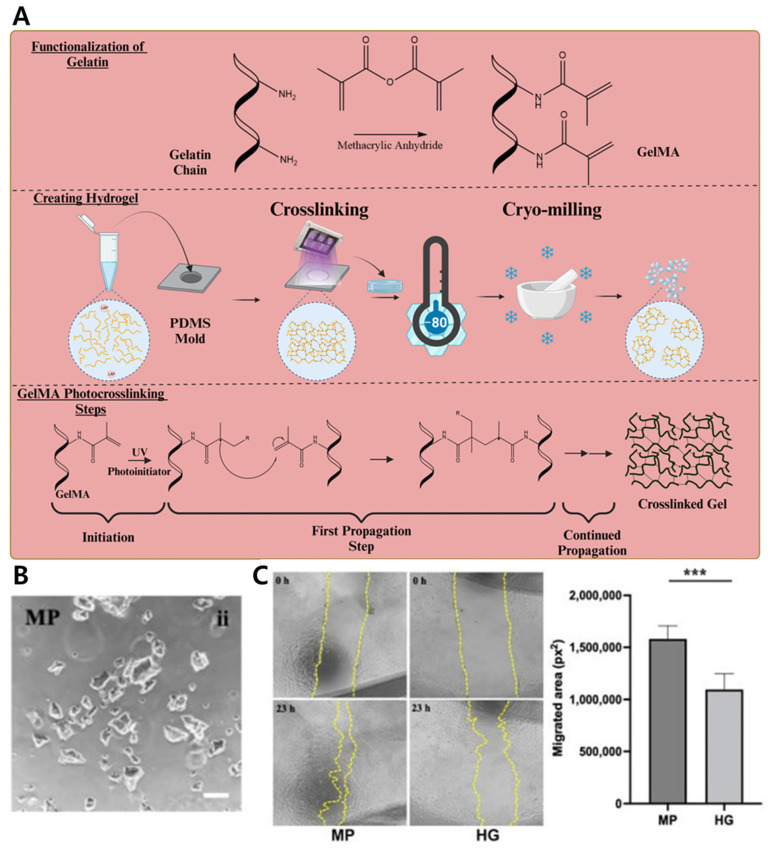
*(***A**) The key steps of making GelMA MPs are illustrated. (**B**) ECHO microscope image of MPs. (**C**) MP scratch wound migration assay image and area quantification. Reprinted with permission from complete reference citation [207]. Copyright © 2025, Molecular Diversity Preservation International.

A hybrid microparticle-based platform was developed to co-deliver human mesenchymal stem cells (hMSCs) and antibiotics for synergistic wound healing. The system utilized gelatin methacrylate (GelMA) microparticles (MPs) produced via microfluidic droplet generation, which served as carriers for both therapeutic agents (Figure 3A). Penicillin-streptomycin (PS) antibiotics were first encapsulated in gelatin nanoparticles using a desolvation method, then embedded within the GelMA MPs along with hMSCs. The MPs were crosslinked with UV light to retain structural integrity while preserving cell viability (Figure 3B). The MP/NP + PS(A) formulation demonstrated dual functionality in vitro, maintaining both antibacterial activity and wound healing potential. In experiments against *S. aureus*, this formulation achieved complete bacterial eradication by day 4 and sustained growth inhibition through day 8. Furthermore, conditioned medium from hMSCs cultured on this formulation significantly enhanced fibroblast migration in a scratch assay, indicating preserved paracrine-driven pro-healing effects (*** *p* < 0.001) (Figure 3C). The compartmentalization of nanoparticles within microparticles enabled spatial segregation of payloads, allowing controlled release kinetics and minimizing cytotoxicity to the encapsulated stem cells. This modular design supports tunable drug-to-cell ratios, injectable delivery, and integration with existing wound scaffolds or dressings, suggesting potential applicability to complex chronic wound environments and disease-associated lesions [207].

### 4.2. Cancer Therapy

#### 4.2.1. Gel–Zein Nanoparticle Composite for Co-Delivery of Doxorubicin and Quercetin in Breast Cancer Cells

**Figure 4:**
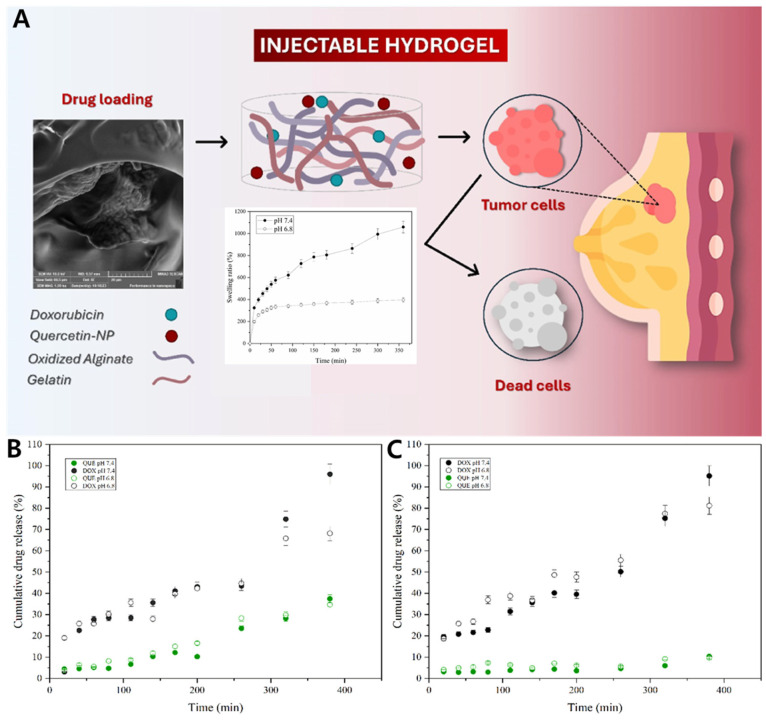
(**A**) Schematic illustration of the injectable dual-drug hydrogel system for breast cancer therapy. (**B**,**C**) Individual and combined comparative drug release profiles of QUE and DOX from hydrogel composites at pH 7.4 and pH 6.8. Reprinted with permission from the complete reference citation [208]. Copyright © 2024, American Chemical Society.

A composite hydrogel system was designed for dual-drug delivery in breast cancer therapy by combining a gelatin–oxidized alginate (Gel–OA) hydrogel matrix with doxorubicin and chitosan-coated zein nanoparticles loaded with quercetin (QNP) (Figure 4A). The hydrogel matrix was prepared by oxidizing alginate to introduce aldehyde groups, which then reacted with the amine groups of gelatin via Schiff base formation to produce an injectable, in situ-forming hydrogel. Zein, a hydrophobic corn protein, was used to fabricate nanoparticles by anti-solvent precipitation, enabling encapsulation of hydrophobic quercetin. Doxorubicin hydrochloride, being hydrophilic, was adsorbed to the nanoparticle surface electrostatically after coating with chitosan. Doxorubicin and QNPs were uniformly dispersed within the Gel–OA hydrogel to form a stable dual-loaded composite. Under tumor-mimicking conditions (pH 6.8), in vitro drug release analysis revealed distinct release profiles for the two drugs: doxorubicin exhibited a faster release profile, while quercetin showed a slower release profile. These differences are likely attributable to their respective loading strategies and diffusion barriers—particularly for quercetin, which was loaded in chitosan-coated zein nanoparticles and subsequently embedded within the hydrogel matrix, thus facing multiple diffusion barriers compared to doxorubicin, which was directly dispersed in the hydrogel (Figure 4B,C). This release pattern enabled temporally coordinated pharmacological actions, with doxorubicin being rapidly released for initial tumor cell killing and quercetin being released more slowly to suppress drug resistance pathways and oxidative stress. Such a release strategy enhanced the overall co-therapeutic efficacy, resulting in a 20.7-fold increase in cytotoxicity compared to doxorubicin alone in MCF-7 breast cancer cells. These findings indicate that the formulation exhibits promising co-therapeutic efficacy at the cellular level and may serve as a valuable foundation for future preclinical studies [208].

#### 4.2.2. Dual-Responsive Injectable Hydrogel with PDA Nanoparticles for Spatiotemporal Release of SN-38

A dual-responsive hydrogel system was developed for localized antitumor therapy by incorporating SN-38-loaded cholesterol-modified micelles and photothermal polydopamine (PDA) nanoparticles into a thermosensitive hydrogel matrix. The hydrogel was based on poly(N-isopropylacrylamide-co-acrylamide) [PNIPAM-co-AAm], which shows an LCST around 35 °C, allowing it to form a semi-solid gel at body temperature. PDA nanoparticles were synthesized via oxidative self-polymerization of dopamine in alkaline buffer and exhibited strong NIR photothermal conversion properties. SN-38, an active metabolite of irinotecan with poor solubility, was encapsulated into cholesterol-conjugated polymeric micelles (Chol–PEG–PCL), which were then dispersed into the hydrogel alongside PDA NPs. Upon intratumoral injection, the hydrogel solidified and enabled passive release of SN-38 under physiological conditions. Upon NIR irradiation, PDA nanoparticles locally heated the matrix, accelerating SN-38 release from micelles via enhanced diffusion and partial hydrogel destabilization. This dual-responsive design enabled spatially confined and temporally controlled drug delivery, enhancing antitumor efficacy while minimizing systemic exposure in an HCT-116 murine colon tumor model [209].

#### 4.2.3. Sequential Local Release of Doxorubicin and Docetaxel Using Thermoresponsive Hydrogel–Micelle Hybrid

A dual-drug delivery system was designed to enhance localized cancer therapy by combining a thermosensitive hydrogel loaded with doxorubicin and docetaxel-encapsulated mixed micelles. The hydrogel matrix was based on Pluronic F127, a triblock copolymer known for its sol-to-gel transition at body temperature, enabling in situ gelation after injection. Doxorubicin was directly incorporated into the hydrogel solution, allowing rapid release upon gelation. Separately, docetaxel was encapsulated in mixed micelles composed of D-α-tocopheryl polyethylene glycol 1000 succinate (TPGS) and Pluronic L121, which were then physically embedded into the hydrogel matrix. Upon intratumoral injection, the system provided a two-phase release: doxorubicin diffused quickly from the hydrogel matrix to achieve immediate cytotoxicity, while docetaxel was gradually released from the micelles over several days, extending the therapeutic window. The formulation demonstrated enhanced suppression of CT26 tumors in BALB/c mice in vivo, compared to single-drug or free-drug formulations, with significantly reduced systemic toxicity. The use of two distinct compartments—hydrogel for hydrophilic doxorubicin and micelles for hydrophobic docetaxel—enabled independent kinetic control and minimized drug–drug interference [210].

#### 4.2.4. Injectable Alginate Complex Hydrogel Loaded with Dual-Drug Nanovectors for Photochemotherapy of Triple-Negative Breast Cancer

**Figure 5:**
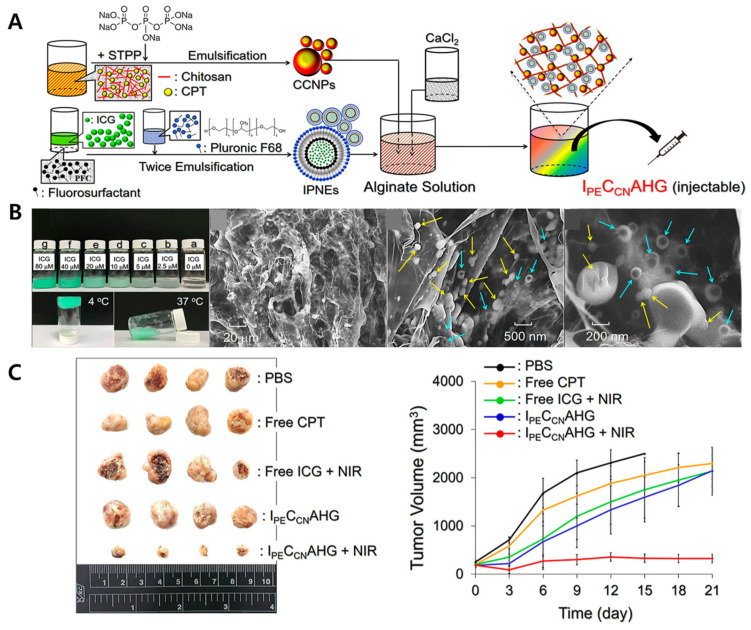
(**A**) Schematic diagram showing the fabrication of CCNPs, IPNEs, and IPECCNAHG. (**B**) Photographs of IPECCNAHG at 4 and 37 °C and SEM images of IPECCNAHG (yellow and blue arrows indicate CCNPs and IPNEs, respectively). (**C**) Photographs and size changes of tumors in all groups that received various treatments for 21 days. Reprinted with permission from the complete reference citation [211]. Copyright © 2024, American Chemical Society.

An injectable alginate-based hydrogel system was developed to deliver two distinct nanocarriers for the treatment of triple-negative breast cancer (TNBC). The hydrogel matrix was formed by crosslinking oxidized alginate with adipic acid dihydrazide, creating a biocompatible and biodegradable scaffold. The first nanocarrier consisted of indocyanine green (ICG) encapsulated within perfluorocarbon nanoemulsions (IPNEs), designed to enhance photothermal and photodynamic therapy upon NIR irradiation. The second nanocarrier comprised camptothecin (CPT) loaded into chitosan nanoparticles (CCNPs) for sustained chemotherapeutic effects (Figure 5A). These nanocarriers were uniformly dispersed within the hydrogel matrix (Figure 5B). Upon intratumoral injection, the hydrogel facilitated localized retention and sequential release of the therapeutic agents. NIR irradiation activated the IPNEs, inducing hyperthermia and reactive oxygen species generation, while the CCNPs provided prolonged CPT release to inhibit tumor cell proliferation. In vivo studies using a murine TNBC tumor model demonstrated significant tumor growth suppression and minimal systemic toxicity (Figure 5C), highlighting the potential of this dual-delivery hydrogel system for effective TNBC therapy [211].

#### 4.2.5. pH-Responsive Chitosan Hydrogel for Co-Delivery of 5-Fluorouracil and Everolimus in Breast Cancer Therapy

**Figure 6:**
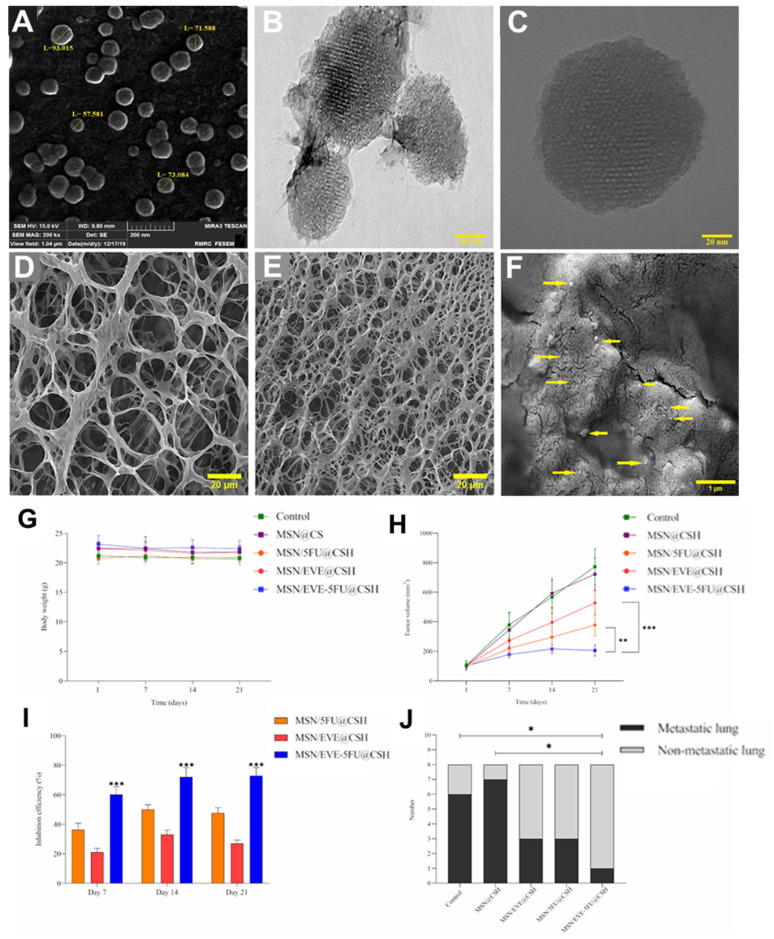
Morphological analysis of MSNs and CSH: (**A**) SEM image and (**B**,**C**) TEM images of MSNs; SEM images of (**D**) pure CSH, (**E**) β-GP-crosslinked CSH and (**F**) β-GP-crosslinked CSH with incorporated MSNs (The yellow arrows indicate the MSNs). In vivo evaluation of the nanosystem in a BALB/c mouse breast cancer model: (**G**) body weight changes and (**H**) tumor volume changes in mice during and after the treatment period. (**I**) Tumor growth inhibition efficiency for each treatment group. (**J**) Number of mice with lung metastases in each group after treatment. Reprinted with permission from the complete reference citation [60]. Copyright © 2025, BioMed Central.

A recent study introduced an injectable, pH-responsive chitosan-based hydrogel system embedded with MSNs for the co-delivery of 5-fluorouracil (5-FU) and everolimus, aiming to enhance breast cancer treatment efficacy. The hydrogel was synthesized by crosslinking chitosan with genipin, forming a biocompatible matrix capable of in situ gelation upon subcutaneous injection near tumor sites (Figure 6A–F). MSNs were functionalized to encapsulate both 5-FU, a chemotherapeutic agent, and everolimus, an mTOR inhibitor, facilitating their co-loading and controlled release. The drug release from the hydrogel was designed to be pH-sensitive, ensuring accelerated release in the acidic tumor microenvironment. In vitro studies demonstrated enhanced cellular uptake and increased apoptosis in 4T1 breast cancer cells treated with the dual-drug-loaded hydrogel compared to monotherapies. In vivo experiments using a Balb/C mouse model showed significant tumor growth inhibition and reduced lung metastasis in groups treated with the co-delivery system (* *p* < 0.05, ** *p* < 0.01, *** *p* < 0.001) (Figure 6G–J). This approach underscores the potential of stimuli-responsive hydrogel–nanoparticle composites in delivering combination therapies for improved cancer treatment outcomes [60].

### 4.3. Infection Control

#### 4.3.1. Efflux Pump Inhibition Combined with Antibiotic Delivery for Resistant Skin Infections

**Figure 7:**
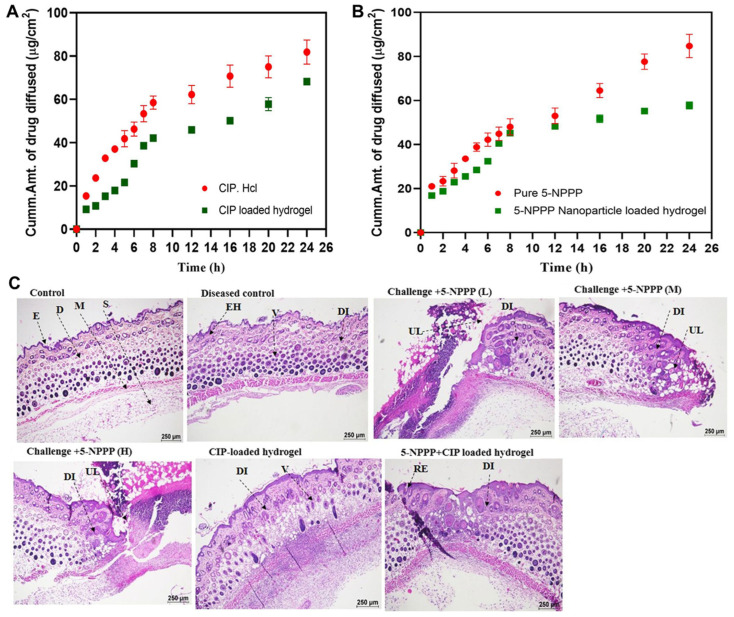
(**A**,**B**) In vitro permeation analysis of drugs from drug-loaded hydrogels in comparison with the permeation of pure drugs. (**C**) Histopathological analysis of skin samples collected for drug efficacy analysis against infection. Reprinted with permission from the complete reference citation [31]. Copyright © 2023, Nature Publishing Group.

A dual-drug delivery system was developed utilizing PVA hydrogels embedded with ciprofloxacin (CIP) and 5-nitrophenylpiperazine (5-NPPP)-loaded Eudragit RSPO nanoparticles to treat Staphylococcus aureus-induced skin infections. CIP, a fluoroquinolone antibiotic, was incorporated directly into the PVA hydrogel matrix, while 5-NPPP, an efflux pump inhibitor targeting NorA, was encapsulated within Eudragit RSPO nanoparticles prepared via a modified nanoprecipitation–solvent evaporation method, achieving particle sizes of 230–280 nm and over 90% drug entrapment efficiency. The dual-drug-loaded hydrogels were fabricated by integrating free CIP and 5-NPPP-loaded nanoparticles into a 7% *w*/*v* PVA solution, followed by crosslinking to form hydrogels with a diameter of approximately 2.2 cm and a thickness of 0.5–0.6 mm. In vitro release studies demonstrated a sustained release of both drugs over five days, with the release kinetics best fitting the Makoid–Banakar and Korsmeyer–Peppas models, indicating diffusion-controlled release mechanisms (Figure 7A,B). The hydrogels were applied topically to Balb/c mice models with *S. aureus*-induced soft skin infections, resulting in significant reductions in bacterial load and re-sensitization of the bacteria to CIP, thereby enhancing the antibiotic’s efficacy (Figure 7C). This approach effectively combines antibiotic therapy with efflux pump inhibition to overcome bacterial resistance mechanisms in localized skin infections [31].

#### 4.3.2. Layered 3D-Printed Hydrogel for Dual Antibiotic Delivery Against Implant-Associated Infections

A dual-drug delivery system was developed using 3D-printed GelMA hydrogels incorporating PLGA nanoparticles loaded with rifampicin (Rif) and vancomycin (Van) to address implant-associated infections caused by antibiotic-resistant Staphylococcus aureus. Rif- and Van-loaded PLGA nanoparticles were synthesized via double- and single-emulsion solvent evaporation methods, respectively, with low-molecular-weight PLGA selected to achieve rapid drug release within the first 7 days. These nanoparticles were embedded into GelMA hydrogels, which were then fabricated into four distinct 3D-printed constructs containing non-loaded nanoparticles, Rif-NPs, Van-NPs, or alternating layers of Rif-NPs and Van-NPs. The layered GelMA hydrogel co-delivering Rif and Van effectively eradicated *S. aureus* strains resistant to either antibiotic, while hydrogels containing only one antibiotic led to the development of resistant colonies due to known mutations, such as in the rpoB gene. This study demonstrates that spatially controlled, dual antibiotic delivery via 3D-printed GelMA hydrogels can prevent the emergence of resistance and effectively combat implant-associated infections [114].

#### 4.3.3. Sequential Triple Antibiotic Release from Hierarchical 3D Bioceramic–Polymer Scaffolds for Biofilm Eradication

A hierarchical 3D scaffold was engineered by integrating a nanocomposite bioceramic core with PVA and an external gelatin-glutaraldehyde (Gel-Glu) coating to achieve spatially controlled, sequential delivery of three antibiotics—rifampicin, levofloxacin, and vancomycin—for the treatment of bone infections. Rifampicin was incorporated into the outer Gel-Glu layer to facilitate rapid initial release, levofloxacin was loaded into the mesoporous structure of the bioceramic core for sustained release, and vancomycin was embedded within the PVA polymer matrix to provide intermediate release kinetics. This compartmentalized design enabled a programmed release profile: an immediate burst of rifampicin to disrupt biofilms, followed by prolonged release of levofloxacin and vancomycin to eliminate residual bacteria. In vitro studies demonstrated that this multidrug scaffold effectively eradicated both Gram-positive and Gram-negative bacterial biofilms, while also supporting preosteoblast adhesion and proliferation across the scaffold surface, indicating its potential for concurrent infection control and bone tissue regeneration [212].

#### 4.3.4. Dual-Responsive Hydrogel Patch for Tailored Antibiotic Release in Chronic Wound Infections

**Figure 8:**
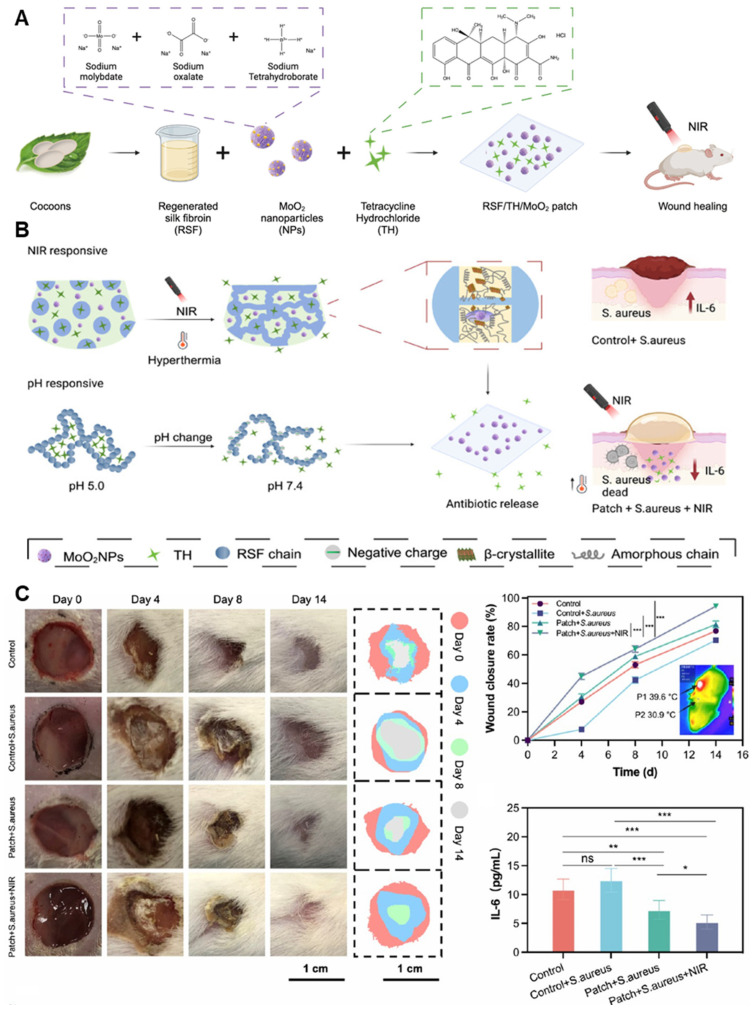
(**A**) Schematic illustration of RSF/TH/MoO_2_NP hydrogel patch synthesis for infected cutaneous wound healing. (**B**) Near-infrared (NIR) and pH-responsive drug release mechanism of the RSF/TH/MoO_2_NPs hydrogel patch. (**C**) The in vivo wound-healing assay was evaluated through time-course photographs, overlaid wound area schematics, and quantitative analysis of closure rates under various treatment conditions including *S. aureus* infection and NIR-assisted hydrogel therapy (* *p* < 0.05, ** *p* < 0.01, *** *p* < 0.001). Reprinted with permission from complete reference citation [213]. Copyright © 2024, American Chemical Society.

A dual-drug delivery hydrogel patch was developed using regenerated silk fibroin (RSF) and molybdenum dioxide (MoO_2_) nanoparticles to treat chronically infected wounds with precise antibiotic release in response to both pH and NIR light (Figure 8A). MoO_2_ nanoparticles were synthesized and dispersed within the RSF hydrogel matrix, enabling photothermal conversion under 808 nm NIR irradiation. Antibiotics were physically loaded into the hydrogel during gelation, with the matrix designed to release more drug under alkaline conditions (pH > 7), characteristic of infected wounds (Figure 8B). NIR exposure further accelerated drug release via localized heating, enhancing penetration and bactericidal effects. The composite hydrogel demonstrated strong mechanical integrity, on-demand antibacterial activity, and biodegradability tailored to the healing process. In murine wound models infected with *S. aureus*, the patch effectively reduced bacterial load and supported tissue regeneration (Figure 8C). Although the RSF/TH/MoO_2_NP-based hydrogel patch was proposed as a platform for chronic wound treatment, its in vivo validation was limited to an acute infection model. The study employed a skin wound model infected with a single bacterial strain (*S. aureus*) only, without incorporating pathological features of chronic wounds such as diabetic conditions, biofilm formation, or delayed healing. Nevertheless, based on its dual responsiveness and excellent biocompatibility, this system holds promise for future application in more complex chronic wound environments [213].

### 4.4. Transplant Immunosuppression

#### 4.4.1. Localized Dual Delivery of Immunosuppressant and Antibiotic via Micelle-Loaded Peptide Hydrogel for Corneal Graft Rejection

A recent study developed a dual-drug delivery system targeting corneal graft rejection by co-delivering rapamycin (RAPA), an immunosuppressant, and levofloxacin hydrochloride (Lev), an antibiotic, using a cationic peptide-based hydrogel (NapFFKK) loaded with Lev@RAPA micelles. RAPA was encapsulated into mPEG-PCL micelles to enhance its solubility and stability, while Lev was directly incorporated into the hydrogel matrix. The resulting composite hydrogel exhibited sustained in vitro release of both drugs without compromising their pharmacological activities. In vitro assays confirmed that RAPA retained its anti-inflammatory effects (via mTOR inhibition and cytokine suppression), and Lev preserved its antibacterial efficacy, comparable to commercial Lev gel^®^, thereby indicating no functional antagonism. Furthermore, in vivo ocular pharmacokinetic studies demonstrated that both drugs maintained independent absorption profiles without mutual interference. This indicates that the composite formulation not only ensures pharmacological stability, but also improves localized therapeutic efficacy by providing sustained immunosuppressive and antibacterial effects at the graft site, thereby reducing the risk of corneal graft rejection and minimizing systemic side effects commonly associated with conventional therapies [83].

#### 4.4.2. Vesicle-Crosslinked Hydrogel for Localized Immune Modulation in Allogeneic Transplantation

**Figure 9:**
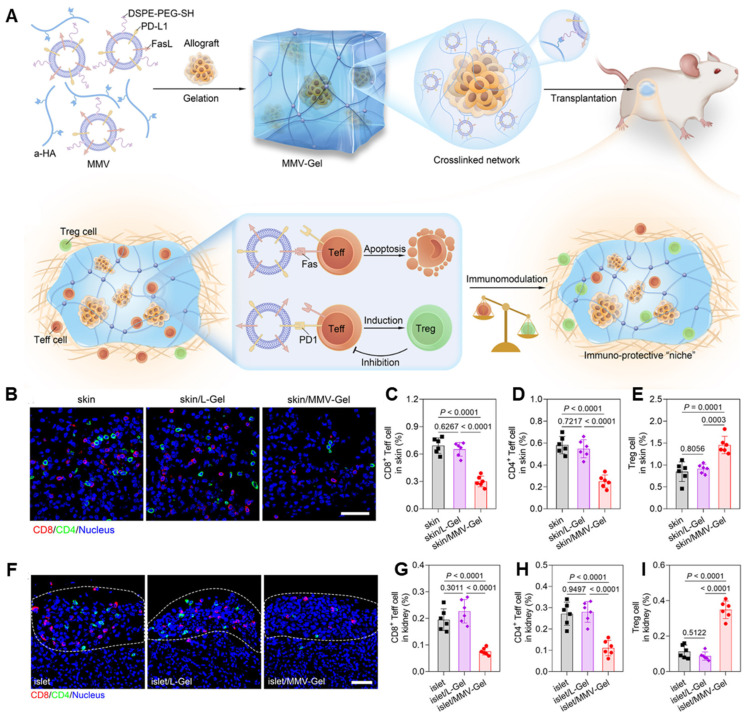
(**A**) Schematic diagram of MMV-Gel preparation for local delivery of allograft and schematic diagram of the mechanism of MMV-Gel. (**B**) Immunofluorescence images showing the infiltration of CD8^+^ and CD4^+^ T cells in skin grafts at day 7 post-transplantation in the skin. (**C**–**E**) Skin/MMV-Gel groups and flow cytometric analysis of CD8^+^ Teff, CD4^+^ Teff, and Treg cell populations (CD8^+^CD44^+^CD62L^−^, CD4^+^CD44^+^CD62L^−^, and CD4^+^FoxP3^+^, respectively) in the transplanted skin. (**F**) Immunofluorescence images showing the infiltration of CD8^+^ and CD4^+^ T cells in the transplanted kidney at day 7 post-transplantation in the islet, islet/L-Gel. (**G**–**I**) Islet/MMV-Gel groups and flow cytometric analysis of CD8^+^ Teff, CD4^+^ Teff, and Treg cell populations (CD8^+^CD44^+^CD62L^−^, CD4^+^CD44^+^CD62L^−^, and CD4^+^FoxP3^+^, respectively) in the transplanted kidney. Reprinted with permission from complete reference citation [214]. Copyright © 2024, Nature Publishing Group.

A recent study introduced a dual-function hydrogel system designed to enhance allograft survival by locally modulating immune responses. The system utilizes a hydrogel crosslinked with mesenchymal stem cell membrane-derived vesicles (MMVs) engineered to overexpress Fas ligand (FasL) and programmed death ligand 1 (PD-L1) (Figure 9A). These vesicles are integrated into the hydrogel matrix, creating an immunosuppressive microenvironment at the transplantation site. Upon implantation in allogeneic islet and skin transplant models, the hydrogel facilitates the induction of T effector cell apoptosis and promotes the generation of regulatory T cells, thereby reducing immune-mediated graft rejection (Figure 9B–I). This localized approach aims to minimize the need for systemic immunosuppression, potentially reducing associated side effects [214].

## 5. Conclusions and Future Perspectives

The advancement of dual-drug delivery systems using hydrogel–nanoparticle composites represents a significant evolution in the field of precision therapeutics. By leveraging the complementary properties of hydrogels and nanoparticles, these systems provide spatial and temporal control over the release of two pharmacologically distinct agents, enabling synergistic or sequential therapeutic actions [215,216]. Hydrogels offer a hydrated three-dimensional matrix that can be engineered for biodegradability, mechanical tunability, and injectability, while nanoparticles serve as vehicles for encapsulating hydrophobic or unstable drugs, often allowing for intracellular delivery or stimuli-responsive release [217,218,219,220]. When these two components are integrated into a composite system, their functionalities not only add but often multiply, resulting in platforms capable of achieving sophisticated release kinetics, site-specific activation, and enhanced bioavailability for both drugs [221,222,223]. This integration is particularly valuable in clinical scenarios where complex pathophysiological processes—such as chronic inflammation, hypoxia, infection, or tumor heterogeneity—demand more than single-agent pharmacology.

In recent years, various fabrication strategies, including emulsion-based nanoparticle encapsulation, in situ crosslinking hydrogels, and microfluidics-enabled architectures, have expanded the structural and functional diversity of these systems [224]. Representative applications in this review have demonstrated the feasibility and therapeutic advantage of dual-delivery approaches in fields such as diabetic wound healing, localized cancer therapy, infection control, and tissue regeneration. These systems often exploit compartmentalization strategies, such as encapsulating one drug in hydrogel matrices and another in embedded nanoparticles, to preserve individual release profiles and minimize drug–drug interference. Moreover, the use of stimuli-responsive materials, including pH-, temperature-, enzyme-, or redox-sensitive elements, allows for the dynamic modulation of drug release in response to the tissue microenvironment. Collectively, the progress in this area affirms that hydrogel–nanoparticle composite systems are not simply carriers but are increasingly becoming programmable delivery devices capable of mimicking or complementing biological processes. The field has moved from proof-of-concept studies to highly tailored systems with clinically relevant performance in animal models. While many challenges remain, the body of evidence supports the viability and translational potential of these platforms as next-generation therapeutic modalities for complex diseases.

Despite the promising advancements in dual-drug delivery using hydrogel–nanoparticle composites, several critical limitations continue to hinder their broader translational potential. One of the most fundamental challenges lies in the precise control of release kinetics for two distinct drugs with inherently different physicochemical properties. Achieving synchronized or sequential release without compromising the activity or stability of either agent remains a formulation challenge, particularly in vivo, where variable pH, enzymatic activity, and interstitial flow can unpredictably alter material behavior [225]. Additionally, co-encapsulation or overlapping release of drugs can lead to unintended interactions, such as antagonistic effects or premature degradation [226]. Ensuring spatial compartmentalization within the hydrogel matrix, while maintaining uniform mechanical properties and injectability, is another nontrivial engineering task [227]. Biocompatibility and long-term safety also pose barriers, especially when synthetic polymers or residual crosslinkers are used [39]. Even when bioinert materials are chosen, their degradation products may lead to inflammatory responses or interfere with tissue regeneration [228]. Manufacturing complexity is another hurdle; integrating nanoparticle synthesis, hydrogel formulation, and sterilization into a scalable and reproducible workflow remains difficult. Moreover, lot-to-lot variability and insufficient standardization in material characterization and drug loading efficiency can compromise batch consistency and regulatory approval [229,230]. From a biological standpoint, most preclinical studies are conducted in rodent models with simplified disease progression, which may fail to capture the complexity of human tissue physiology, immune responses, and pharmacokinetics. Lastly, regulatory pathways for combination products involving multiple active pharmaceutical ingredients, nanomaterials, and hydrogel scaffolds are still evolving and often lack clear guidelines, making clinical translation an uncertain and prolonged process [231,232,233].

Looking ahead, the future development of dual-drug delivery systems using hydrogel–nanoparticle composites will likely be driven by several converging technological and clinical trends. One critical direction is the refinement of multi-stimuli-responsive systems that can respond to combinations of endogenous (e.g., pH, redox, enzyme) and exogenous (e.g., light, temperature, magnetic fields) triggers. Such designs allow for layered, temporally adjustable, or feedback-controlled release, enabling better alignment with dynamic disease microenvironments. The integration of machine learning and predictive modeling into drug formulation design is also rapidly advancing, providing powerful tools for optimizing formulation composition, release kinetics, and therapeutic outcomes. By leveraging large datasets from in vitro and in vivo release studies, AI-driven approaches can help identify optimal material properties and drug loading ratios, enabling the development of personalized or disease-specific release profiles [234,235]. At the same time, progress in microfluidics, 3D bioprinting, and in situ gelation technologies opens up new possibilities for generating patient-specific constructs with spatially defined drug compartments, potentially enabling real-time customization of composite carriers at the point of care [236,237,238]. Another important frontier is the expansion of therapeutic targets beyond cytotoxic or antimicrobial agents. Combining immunomodulators, anti-fibrotic drugs, metabolic regulators, or RNA-based therapeutics within a single composite scaffold could allow simultaneous modulation of immune response, tissue repair, and cellular reprogramming [239,240]. In particular, immunoengineered hydrogels capable of locally delivering both immune checkpoint inhibitors and tumor antigens are gaining attention for their potential to convert non-responsive tumors into immunologically active sites [241]. Furthermore, regulatory science may need to adapt to address the hybrid nature of these systems, possibly by introducing a new classification—such as “multi-agent programmable scaffolds”—that bridges the gap between conventional drug–device combinations and tissue-engineered products. As our understanding of disease heterogeneity deepens and the demand for precision medicine intensifies, hydrogel–nanoparticle composite systems stand poised to play a pivotal role not only as drug depots, but as intelligent, adaptable platforms for coordinated, multimodal therapy.

## Figures and Tables

**Table 2:** Summary of representative hydrogel–nanoparticle composite systems for biomedical applications.

Key Biomedical Applications	Used Hydrogel	Used NPs	Formulation Strategy	Authors
Wound Healing	N-carboxyethyl chitosan Oxidized sodium alginate	Mesoporous polydopamine	Physical Embedding	Tan et al. [204]
	Chitosan	Magnetic montmorillonite	Physical Embedding	Sayyar et al. [205]
	Sodium alginate	Chitosan	Physical Embedding	Mousavi et al. [206]
	Polyethylene glycol diacrylate Thiolated alginate	Chitosan Gelatin	Layer-by-Layer Assembly	Zhang et al. [54]
	Gelatin methacrylate	Gelatin	Physical Embedding	Winkler et al. [207]
Cancer Therapy	Oxidized alginate Gelatin	Chitosan	Physical Embedding	Nascimento et al. [208]
	Poly(n-isopropylacrylamide)	Polydopamine Cholesterol	Covalent Integration Physical Embedding	Liu et al. [209]
	Pluronic F127	Pluronic F127 Pluronic L121	Physical Embedding	Sheu et al. [210]
	Sodium alginate	Pluronic F68 Chitosan	Physical Embedding	Lee et al. [211]
	Chitosan	Mesoporous silica	Physical Embedding	Arvejeh et al. [60]
Infection Control	Polyvinyl alcohol	Eudragit RSPO	Physical Embedding	Thamilselvan et al. [31]
	Gelatin methacrylate	PLGA	Layer-by-Layer Assembly	Martínez-Pérez et al. [114]
	Pluronic F127 Polyvinyl alcohol Gelatin–glutaraldehyde	Apatite	Layer-by-Layer Assembly	García-Alvarez et al. [212]
	Silk fibroin	Molybdenum dioxide	Physical Embedding	Guo. et al. [213]
Transplant Immunosuppression	Cationic peptide	Methoxy poly(ethylene glycol)-poly(ε-caprolactone)	Physical Embedding	Xu et al. [83]
	Thiol-terminated PEGylated phosphatidylethanolamine	Acylated hyaluronic acid	Covalent Integration	Wang et al. [214]

## Data Availability

The datasets generated during and/or analyzed during the current study are available from the corresponding author on reasonable request.

## Abbreviations

The following abbreviations are used in this manuscript:

ROS	Reactive oxygen species
LBL	Layer-by-layer
DDS	Drug delivery system
PEG	Polyethylene glycol
PVA	Polyvinyl alcohol
PNIPAM	Poly(N-isopropylacrylamide)
PEGDA	Polyethylene glycol(PEG)-diacrylate
LCST	Lower critical solution temperature
PLGA	Polylactic-co-glycolic acid
PLA	Polylactic acid
AuNPs	Gold nanoparticles
MSNs	Mesoporous silica nanoparticles
NIR	Near-infrared
MMP	Matrix metalloproteinase
GSH	Glutathione
MPDA	Mesoporous polydopamine
MMT	Montmorillonite
CS	Chitosan
CSNPs	Chitosan nanoparticles
PGMs	Pirfenidone-loaded gelatin microspheres
Cur	Curcumin
hMSCs	Human mesenchymal stem cells
GelMA	Gelatin methacrylate
MPs	Microparticles
PS	Penicillin–streptomycin
OA	Oxidized alginate
QNP	Nanoparticles loaded with quercetin
PDA	Polydopamine
TPGS	D-α-tocopheryl polyethylene glycol 1000 succinate
TNBC	Triple-negative breast cancer
ICG	In-docyanine green
IPNEs	In-docyanine green-loaded perfluorocarbon nanoemulsions
CPT	Camptothecin
CCNPs	Camptothecin-loaded chitosan nanoparticles
SEM	Scanning electron microscopy
TEM	Transmission electron microscopy
CSH	Chitosan hydrogel
β-GP	Beta-glycerophosphate
CIP	Ciprofloxacin
5-NPPP	5-nitrophenylpiperazine
Rif	Rifampicin
Van	Vancomycin
Gel-Glu	Gelatin–glutaraldehyde
RSF	Regenerated silk fibroin
MoO_2_	Molybdenum dioxide
RAPA	Rapamycin
Lev	Levofloxacin hydrochloride
NapFFKK	Cationic peptide-based hydrogel
MMVs	Membrane-derived vesicles
